# Impacts of Stressful Life Events and Traumatic Experiences on Onset of Obsessive-Compulsive Disorder

**DOI:** 10.3389/fpsyt.2020.561266

**Published:** 2020-12-03

**Authors:** Keitaro Murayama, Tomohiro Nakao, Aikana Ohno, Sae Tsuruta, Hirofumi Tomiyama, Suguru Hasuzawa, Taro Mizobe, Kenta Kato, Shigenobu Kanba

**Affiliations:** ^1^Department of Neuropsychiatry, Graduate School of Medical Sciences, Kyushu University, Fukuoka, Japan; ^2^Graduate School of Human-Environment Studies, Kyushu University, Fukuoka, Japan; ^3^Karatsu Red Cross Hospital, Karatsu, Japan

**Keywords:** type of symptoms, onset, traumatic experiences, stressful life events, obsessive-compulsive disorder (OCD)

## Abstract

Not a few patients with obsessive-compulsive disorder (OCD) have experienced events that affected the onset. The onset of OCD is not limited to the original meaning of trauma; rather, traumatic experiences such as unexpected exposure to contaminants or various stressful life events often cause the onset of OCD. It would be useful to understand the experiences surrounding the onset, including stressful life events and traumatic experiences, for comprehension of the pathophysiology of OCD. In the present study, we investigated the onset conditions of 281 patients with OCD and compared clinical characteristics among groups with or without stressful life events including traumatic experiences. As a result, 172 (61.2%) participants had experienced various stressful life events, and 98 (34%) participants had had traumatic experiences before the onset. Furthermore, the participants who had had stressful life events showed more contamination/fear symptoms compared with those without such life events. Meanwhile, the patients who had had specific traumatic experiences showed a tendency toward hoarding obsessions. To comprehend the pathophysiology of OCD, it is important to understand the stressful life events that precede its onset.

## Introduction

Obsessive-compulsive disorder (OCD) is characterized by persistent intrusive thoughts and repetitive actions, significantly affecting the patient's daily life. Most patients with OCD show comparatively early onsets, i.e., during their adolescence. There is a difference in onset peaks between males and females, with male patients having the peak in the mid-teenage years and female patients in the mid-twenties. This suggests that male OCD is affected strongly by genetic factors and comorbid neurodevelopmental disorders such as tic and autistic spectrum disorders, while female OCD is influenced by comorbid mood or anxiety disorders and life events such as marriage, pregnancy, and childbirth (1). From the aspect of cognitive behavioral theory, patients with OCD were fear conditioned by stimulants typical of the subject of the obsession at its onset. Subsequently, their avoidant behaviors, including compulsions, were maintained because those behaviors temporally alleviated their anxieties and fears. Mowrer described this process as a “two-process theory of learning” (2).

Furthermore, not a few patients with OCD experienced episodes that affected the onset of their OCD. In addition to the original meaning of trauma, stressful life events or unexpected exposure to contaminants often are associated with the onset of OCD (3, 4). In addition, Vidal-Ribas et al. reported that experiences of abuse, sexual abuse, and family disruption were associated with the severity of obsessive-compulsive symptoms (5). In cases of OCD, while stressful life events and traumatic experiences cause the onset of obsessive symptoms directly, compulsive behaviors caused by these experiences maintain and gradually worsen the symptoms. Then, to what extent do these experiences and events affect the onset of OCD? Recently, Rosso et al. found that around 60% of 329 participants in a study developed OCD after experiencing stressful life events (6). Another study reported that around 50% of OCD patients had experienced at least one traumatic life event in their lifetime (7). Bogetto et al. suggested that women may have greater risk of initial onset of OCD after precipitating events (8). In the most recent study which included 954 OCD outpatients, Destrée et al. reported that termination of a relationship was associated with a faster speed of progression to OCD (9). In our previous study (10) that examined 36 patients with OCD, over 60% patients also experienced either stressful life events within 1 year or trauma within 1 month, respectively, before the onset. The results also suggested that most cases of OCD that experienced a traumatic incident before the onset were male children with contamination/fear symptoms. Furthermore, while female patients experienced stressful life events including family problems, pregnancy, and childbirth, male patients mostly reported experiences of changing jobs or relocating before the onset.

In the present study, to comprehend the pathophysiology of OCD, we examined a large sample of patients with OCD to identify the conditions around the onset, including stressful life events and traumatic experiences. First, we divided the participants into two groups (with or without stressful life events including traumatic experiences) around the time of the OCD onset. In addition, we identified a traumatic subgroup experiencing trauma within 1 month before the onset. To understand the relationship between onset conditions and the pathophysiology of OCD, we compared the clinical information and characteristics of these groups.

## Methods and Materials

Participants were recruited from outpatients of the Department of Psychiatry of Kyushu University Hospital from January 2014 to December 2018. The inclusion criterion was that they meet the OCD diagnostic criteria of DSM-5. In this study, after acquiring informed consent, psychiatrists who specialize in OCD treatment conducted psychiatric interviews with the participants to obtain clinical information that included the background, comorbid condition(s), history of tic episodes, and past/current OCD symptoms. The global symptom severity of OCD patients was assessed using the Yale-Brown Obsessive-Compulsive Scale (Y-BOCS) (11, 12). The patients also completed the Hamilton Depression Rating Scale (HDRS) (13) and Hamilton Anxiety Rating Scale (HARS) (14) to assess the severity of depression and anxiety. OCD patients who had a history of neurological illness, other disorders of the central nervous system, or a history of substance abuse were excluded.

We defined events such as an entrance examination, marriage, childbirth, economic problems, occupational problems, personal relationship problems which were experienced within 1 year prior to the onset of OCD as “stressful life events.” Our questionnaire of the typical life events was cited from *Scaling for Life Events* by Paykel et al. (15). We adopted a semistructured, clinician-administered interview for this study. First, we asked the patients to recall the age at which symptoms of OCD first began. Second, the participants were also asked any stressful life events, referring the questionnaire, experienced within 1 year prior to the onset OCD. If there were other stressful life events within the period, participants also could describe them to the interviewer. Furthermore, we asked the participants about the traumatic event in detail. We defined traumatic experiences as those that were experienced within 1 month before the onset of OCD and had a strong impact on its onset directly.

The studies involving each participating patient were reviewed and approved by the Ethics Committee of the Kyushu University (No. 2019-216).

## Results

Two hundred eighty-one participants with OCD met the inclusion criteria of this study. Their ages ranged from 18 to 65 years. The participants were divided into two groups including a stressful life event-related OCD group (LE-OCDs) and a spontaneous-onset OCD group (Sp-OCDs) based on the presence or absence of stressful life events. Furthermore, we identified a subgroup of the LE group having traumatic experiences within 1 month before the onset (TE-OCDs). Demographic and clinical findings of the LE- or TE-OCDs and Sp-OCDs were compared. A two-tailed group *t*-test was used to determine statistical significance. For categorical data (i.e., sex, marital status, comorbidities), a chi-square test was used.

Table 1 shows the clinical background of each group. Around 60% of the participants (LE-OCDs, *n* = 172) experienced a stressful life event before the onset of OCD. In addition, 56.9% of LE-OCDs had experienced traumatic events within 1 month prior to the onset (TE-OCDs, *n* = 98). LE-OCDs showed a significantly later onset age (*p* = 0.007) and a higher rate of marriage (*p* = 0.008) compared with Sp-OCDs.

**Table 1:** Clinical background of LE-OCD/TE-OCD vs. Sp-OCD.

	**LE-OCDs**	**TE-OCDs**	**Sp-OCDs**	**Statistics (LE vs. Sp)**				**Statistics (TE vs. Sp)**			
				***X*^**2**^**	***t***	***df***	***p***	***X*^**2**^**	***t***	***df***	***p***
Number (%)	172 (61.2)	98 (34.8)	109 (38.7)								
Sex: M/F (% male)	77/95 (44.8)	42/56 (42.9)	55/54 (50.5)	0.33		1	0.622	0.52		1	0.819
Age: mean (range, SD)	35.1 (15–78, 12.8)	34.3 (15–70, 11.8)	33.3 (13–77, 14.1)		0.52	278	0.600				
Onset age: mean (SD)	26.6 (8–68, 13.2)	25.9 (10–68, 11.8)	21.2 (4–72, 11.4)		2.74	277	0.007		2.83	203	0.005
Educational year: mean (SD)	13.4 (2.5)	13.4 (2.3)	12.5 (2.5)		2.41	276	0.017		2.75	202	0.006
Marital status: married/single or separated (% married)	71/101 (41.3)	40/58 (40.8)	29/80 (26.6)	9.65		2	0.008	4.69		1	0.003
Employed: yes/no (%)	61/111 (35.5)	36/62 (36.7)	34/75 (31.2)	0.05		1	0.826	0.66		2	0.716
Family history of OCD: yes/no (%)	12/160 (7.0)	11/87 (11.2)	4/105 (3.7)	1.36		1	0.244	5.51		1	0.019
Y-BOCS obsession (%)											
Aggression	73 (42.4)	40 (40.8)	32 (39.5)	0.16		1	0.689	0.40		1	0.841
Contamination	110 (64.0)	67 (68.4)	54 (49.5)	5.70		1	0.019	7.53		1	0.006
Sexual	6 (3.5)	4 (4.1)	4 (3.6)	0.01		1	0.589	0.24		1	0.878
Hoarding	24 (12.2)	16 (16.3)	9 (8.2)	2.09		1	0.184	3.17		1	0.075
Religious	21 (12.2)	10 (10.2)	9 (8.2)	1.09		1	0.296	0.24		1	0.628
Symmetry/exactness	32 (18.6)	16 (16.3)	26 (23.9)	1.12		1	0.294	1.81		1	0.179
Somatic	15 (8.7)	10 (10.2)	6 (5.5)	0.07		1	0.806	0.01		1	0.541
Miscellaneous	77 (44.8)	42 (42.9)	47 (43.1)	1.00		1	0.361	1.60		1	0.206
Y-BOCS compulsion (%)											
Cleaning	110 (64)	44 (44.9)	57 (52.3)	3.76		1	0.062	4.85		1	0.028
Checking	83 (48.8)	48 (49.0)	49 (45.0)	0.4		1	0.525	0.34		1	0.562
Repeating	51 (29.7)	21 (21.4)	38 (34.9)	0.84		1	0.360	4.57		1	0.033
Counting	7 (4.1)	4 (4.1)	5 (4.6)	0.04		1	0.834	0.03		1	0.859
Ordering/arranging	20 (11.6)	10 (10.2)	12 (11.0)	0.03		1	0.874	0.04		1	0.851
Hoarding/collecting	18 (10.5)	14 (14.3)	8 (7.3)	0.78		1	0.378	2.62		1	0.105
Miscellaneous	49 (28.5)	26 (26.5)	30 (27.5)	0.03		1	0.861	0.03		0	0.873
Total Y-BOCS score: mean (SD)	26.8 (5.9)	26.5 (6.0)	25.6 (8.4)		0.54	262	0.587	0.88		193	0.383
HAM-A: mean (SD)	4.95 (5.5)	5.16 (6.25)	4.89 (5.5)		5.82	211	0.738		5.45	158	0.437
HAM-D: mean (SD)	6.90 (5.70)	6.97 (5.81)	6.79 (5.72)		4.26	211	0.525		4.87	158	0.814
GAF: mean (SD)	51.82 (6.7)	52.84 (9.3)	51.12 (8.1)		0.57	211	0.571	1.24		158	0.216
Comorbidities											
Mood disorder	22 (12.8)	13 (13.3)	14 (12.8)	0.05		1	0.831	0.18		1	0.672
Anxiety disorder	9 (5.2)	6 (6.1)	8 (7.3)	0.052		1	0.470	0.01		1	0.905
PTSD/acute stress disorder	1 (0.6)	1 (1.0)	0 (0)	0.64		1	0.425	1.26		1	0.262
Neurodevelopmental disorder	18 (10.5)	12 (12.2)	14 (12.8)	0.37		1	0.541	0.04		1	0.846
Other disorder	17 (9.8)	8 (8.2)	19 (17.4)	3.40		1	0.065	3.91		1	0.048
Personality disorder	3 (1.7)	3 (3.1)	3 (2.8)	0.32		1	0.569	0.017		1	0.895
Past history of tic disorder: yes/no (%)	13/149 (13.4)	9/89 (9.2)	15/94 (13.8)	0.01		1	0.926	2.009		2	0.366

*HAM-A, Hamilton Anxiety Rating Scale; HAM-D, Hamilton Depression Rating Scale; LE-OCDs, life event-related OCD group; PTSD, posttraumatic stress disorder; Sp-OCDs, spontaneous onset OCD groups; TE-OCDs, a subgroup of the life event-related OCD group having traumatic experienced within 1 month before the onset; Y-BOCS, Yale Brown Obsessive-Compulsive Scale*.

Concerning Y-BOCS symptoms, LE-OCDs showed significantly more contamination/fear symptoms compared with patients with Sp-OCDs (*p* = 0.019). Furthermore, TE-OCDs showed a tendency toward hoarding obsessions compared with Sp-OCDs (*p* = 0.075). There was no difference between LE-OCDs/TE-OCDs and Sp-OCDs in the severity of Y-BOCS symptoms. There was no difference between these groups in the rates of comorbid mental illness.

Tables 2, 3 show the types of life events and concrete examples of traumatic experiences.

**Table 2:** Types of life events and traumatic experiences.

**Life events related to**	***N* (%)**
Family members	33 (19.19)
Friends	8 (4.65)
Schoolwork	28 (16.27)
Work	31 (18.82)
Aspects of living environment	39 (22.67)
Financial problems	0 (0)

**Table 3:** Details of traumatic experiences.

Contamination	Touched a sticky laundry pole
	Plucked a mobile-phone from a toilet bowl
	Saw a child pick his nose and put it to a train seat
	Saw a person leaking feces
	Saw a coworker handling his white coat like a dirty thing at the hospital
	Worked with infectious waste in a health center
Illness and injury	Got disease of the uterus
	Injured by a chisel
Human relationships	Physical contact with a disliked person
	Scolded by a teacher, teased by classmates
Violence/sexual violence	Was sexually molested
	Treated violently by alcohol-dependent or gambling father
	Abandoned by mother because of father's domestic violence
	Caused a traffic accident
	Pet was killed by somebody
	Began to keep a rabbit as a pet
	Homosexual experience with twin brother
	Treasured doll's head came off
	Smoked marijuana at instigation of an acquaintance
	Watched pornography on the Internet
	Touched a tile which had been exposed to a nuclear bomb

## Discussion

In the present study, we examined the circumstances around the onset of OCD including stressful life events and traumatic experiences of 281 patients. Around 61% patients (LE-OCDs) had experienced stressful life events, including traumatic experiences, before the onset. The result was similar to those of previous studies (6, 7). In addition, around 57% of LE-OCDS patients had traumatic experiences within 1 month before the onset (TE-OCDs). By comparing LE-OCDs with Sp-OCDS, we found that LE-OCDs showed contamination/fear symptoms significantly. Furthermore, TE-OCDs showed a tendency toward more hoarding obsessions compared with Sp-OCDs. This study clarified the impacts of stressful life events and traumatic experiences on the onset of OCD with a comparatively large sample.

### Stressful Life Events and Onset of OCD

Certain patients were exposed to stressful life events before the onset of OCD, even though the events were not critical enough to be considered traumatic experiences. A study (3) examining juvenile patients with OCD found that they experienced more stressful life events within 1 year of the onset compared with healthy controls and patients with other anxiety disorders (3). Furthermore, Neziroglu et al. reported that 29 of 59 married female patients developed OCD during pregnancy (16). Maina et al. found that patients with OCD had experienced childbirth at a significantly higher rate compared with healthy controls (17). These findings showed that the life events of pregnancy and childbirth may be related to the onset of OCD in adult women. Recently, another study examining the relationship between the onset of OCD and stressful life events found that 200 (60.8%) of 329 patients with OCD developed OCD after experiencing stressful life events. More of these patients were female, had an acute onset, and had a physical-related obsession (6).

In the present study, around 61% of the LE-OCD participants had experienced some kind of stressful life event such as family problems, pregnancy, childbirth for females, and changing or relocating of jobs for males within 1 year before the onset. Compared with patients with Sp-OCDs, LE-OCDs showed contamination/fear symptoms significantly. Real et al. reported that there was a positive association between the contamination/cleaning dimension and the onset of OCD close in time to a stressful life event (18). Our result also suggested that the experience of stressful life events might increase the onset risk of OCD, particularly of the contamination/cleaning dimension.

### Traumatic Experiences Causing OCD Symptoms Directly

Patients with OCD show diverse symptoms such as washing rituals with contamination fear, checking rituals with aggressive thoughts, symmetry/ordering, or sexual/religious ideation. Not a few patients experience diverse incidents as a “trigger” before the onset of OCD. In our previous study (10), some patients had direct incidents such as “running over a cat with his car,” “splashing toilet water while assisting a family member,” “classmates letting an insect run over her,” or “making a mistake on her job.” Other patients had triggers that were not serious incidents but subjectively shocking such as “having a disliked classmate touch his game machine” or “lending a pencil that became contaminated with a classmate's sweat.”

Similarly, the participants with traumatic experiences (TE-OCDs) showed various experiences in the present study (Tables 2, 3). Some patients had direct incidents such as “plucked a mobile phone out of a toilet bowl,” “being injured with a chisel,” “causing a traffic accident,” or “having a pet killed by somebody.” Other patients had triggers that were not serious incidents but were subjectively shocking, such as “seeing a child pick his nose and put it on a train seat,” “being touched by a disliked person,” or “seeing pornography on the internet.” Grabe et al. reported that there was no significant association of traumatic experiences with subclinical PTSD or PTSD in patients with OCD compared with controls (19). Actually, though most of these incidents do not meet the original meaning of trauma, they had enough impact to cause the onset of OCD. Therefore, these incidents are “traumatic” for the patients with OCD.

Fontenelle et al. conducted an epidemiological study of 1,001 patients with OCD and reported that 19% of them had comorbid PTSD (20). They also found that posttraumatic OCD is characterized by high rates of late-onset aggression/sexual/religious/hoarding symptoms and comorbid anxiety/mood disorders. Their finding showed that patients with late-onset OCD often had experienced traumatic incidents compared with early-onset, genetic OCD. Meanwhile, we speculated that patients with early-onset OCD also developed their illness after traumatic incidents because of their neurodevelopmental traits and low tolerance for stress. Actually, in our previous study (10) and another Japanese study (21), most cases that experienced a traumatic incident before the onset of OCD were male children with contamination/fear symptoms. While some of them experienced direct exposure to contaminants, the others felt subjectively dirty and disgusted when a disliked person touched them. It is suggested that some patients with contamination/washing are conditioned with disgust, while patients with OCD are generally conditioned with anxiety or fear. Patients with contamination/washing symptoms, therefore, often develop OCD after traumatic experiences. Concerning the association of onset with trauma, the theory of preparedness proposed by Seligman (22) could explain our hypothesis. Seligman suggested that individuals with high preparedness could be conditioned by just one trial, and that the conditioning could become very strong (23). Because most juvenile OCD patients have neurodevelopmental disorder and show low tolerance for stress and lack experience in controlling disgust stimuli, they could be easily and strongly conditioned with a low impact trauma.

In addition, though the difference was not statistically significant, the TE-OCDs showed more hoarding obsessions. Prezeworski et al. reported that patients who had hoarding symptoms alone had experienced higher rates of physical assault and transportation accidents prior to symptom onset than those with OCD symptoms alone (23). These results suggested that hoarding may have a close relationship to traumatic events.

In conclusion, a certain number of patients with OCD had traumatic incidents at the onset, but the contents and severities of their traumas are diverse. Although their experiences do not meet the standard definition of trauma, they can affect the onset of OCD to no small extent. Because OCD has a diversity of etiologies including early onset with neurodevelopmental factors and late onset with cognitive factors, there might be large individual differences in tolerances of and responses to traumatic experiences.

### Limitations

The present study has several limitations. Though we employed large samples of OCD patients and diagnosed them by detailed clinical interviews, we could not perform structured interview for DSM-IV (SCID) for their diagnosis. For the same reason, because we could not conduct SCID for comorbid disorders, the comorbidity ratios were generally low. Second, we did not define the objective criteria for traumatic experiences. Third, the current study had a recall bias since traumatic incidents and stressful life events were identified by retrospective interview. The data we obtained, therefore, have a possibility of factual inaccuracy. Fourth, there might be Berkson's bias since the participants were recruited from the university hospital. Fifth, we did not collect the quantitative data of the stressful life events and the traumatic experiences. We, therefore, could not perform regression analysis to clarify the relationship between the stressful life events and the onset OCD. Sixth, this study did not show the information about treatment implication associated with stressful life event and OCD. Gershuny et al. (24) reported that refractory OCD patients has been associated with the presence of a history of trauma. Alemany-Navarro et al. (25), meanwhile, founded that stressful life events at onset were not the predictor of treatment response for SRIs. Future research will be necessary to elucidate the effect of the environment at onset OCD for the treatment response.

## Conclusion

We examined patients with OCD to identify the circumstances around the onset, including traumatic experiences and stressful life events. We found that around 61% of patients experienced stressful life events before the onset, and around 57% of patients had traumatic experiences within 1 month before the onset. We also found that patients with stressful life events showed significantly more contamination/fear symptoms, and patients with traumatic experiences showed a tendency toward more hoarding obsessions compared with patients with spontaneous onset. Even though these events were not critical situations of real trauma in its original meaning, patients subjectively experienced these events as real “trauma” with intense distress. Since conditioned anxieties and fears are worsened by performing compulsive behaviors in OCD, it would be better to intervene at an earlier time. Furthermore, full understanding of the onset situation will bring good therapeutic cooperation and effective intervention.

## Data Availability Statement

The raw data supporting the conclusions of this article will be made available by the authors, without undue reservation.

## Ethics Statement

The study was approved by an ethics committee of Kyushu University (no. 2019-216), and each participating patient provided written informed consent after receiving a complete description of the study.

## Author Contributions

KM collected the data and wrote the initial draft of the manuscript. TN designed the study and critically reviewed the manuscript. AO and ST contributed to analysis and interpretation of data. HT, SH, TM, and KK have contributed to data collection. SK critically reviewed the manuscript. All authors approved the final version of the manuscript.

## Conflict of Interest

The authors declare that the research was conducted in the absence of any commercial or financial relationships that could be construed as a potential conflict of interest.
